# Recognition, Perceptions and Treatment Practices for Severe Malaria in Rural Tanzania: Implications for Accessing Rectal Artesunate as a Pre-Referral

**DOI:** 10.1371/journal.pone.0000149

**Published:** 2007-01-17

**Authors:** Marian Warsame, Omari Kimbute, Zena Machinda, Patricia Ruddy, Majaha Melkisedick, Thomas Peto, Isabela Ribeiro, Andrew Kitua, Goran Tomson, Melba Gomes

**Affiliations:** 1 Division of International Health (IHCAR), Karolinska Institutet, Stockholm, Sweden; 2 Rectal Artesunate Study Team, National Institute for Medical Research (NIMR), Dar es Salaam, Tanzania; 3 Freelance Consultant in Health Communication, Kampala, Uganda; 4 United Nations Development Programme (UNDP)/World Bank/WHO Special Programme for Research and Training in Tropical Diseases (TDR) Geneva, Switzerland; 5 National Institute for Medical Research (NIMR), Dar es Salaam, Tanzania; Faculty of Tropical Medicine, Mahidol University, Thailand

## Abstract

**Objectives:**

Preparatory to a community trial investigating how best to deliver rectal artesunate as pre-referral treatment for severe malaria; local understanding, perceptions of signs/symptoms of severe malaria and treatment-seeking patterns for and barriers to seeking biomedical treatment were investigated.

**Methodology/Principal Findings:**

19 key informant interviews, 12 in-depth interviews and 14 focus group discussions targeting care-givers, opinion leaders, and formal and informal health care providers were conducted. Monthly fever episodes and danger signs or symptoms associated with severe malaria among under-fives were recorded. Respondents recognized convulsions, altered consciousness and coma, and were aware of their risks if not treated. But, these symptoms were perceived to be caused by supernatural forces, and traditional healers were identified as primary care providers. With some delay, mothers eventually visited a health facility when convulsions were part of the illness, despite pressures against this. Although vomiting and failure to eat/suck/drink were associated with malaria, they were not considered as indicators of danger signs unless combined with another more severe symptom. Study communities were familiar with rectal application of medicines.

**Conclusions/Significance:**

Communities' recognition and awareness of major symptoms of severe malaria could encourage action, but perceptions of their causes and poor discrimination of other danger signs – vomiting and failure to feed – might impede early treatment. An effective health education targeting parents/guardians, decision-makers/advisors, and formal and informal care providers might be a prerequisite for successful introduction of rectal artemisinins as an emergency treatment. Role of traditional healers in delivering such medication to the community should be explored.

## Introduction

Most malaria deaths occur in children in rural areas of sub-Saharan Africa [1]. A major cause is lack of access to prompt effective treatment. Those that present with severe malaria have short histories of illness, emphasizing the speed of disease progression if not treated promptly and effectively [2]. For those children who manage to reach health facilities, hospital based data indicates that the episode commences with a febrile illness 1–3 days prior to admission, and neurological manifestations within 12 hours of admission [3], [4]. There is therefore a brief opportunity (0–16 hours) for therapeutic intervention - to prevent *P. falciparum* parasites from maturing to the more pathogenic sequestered stages, organ failure, and high risk of death (15–20%) for hospital admissions [5].

Reviews of research conducted to understand people's perception and treatment of fever - the primary symptom of malaria and other childhood illnesses [6], [7], have established that acute febrile illness tends to be managed hierarchically- initially convenience and cheaper options dominating before other alternatives are explored. Research has concentrated on febrile malaria - which is common. We know far less through research about how families manage severe malaria which carries a higher mortality, except that convulsions tend to be perceived as a different disease with a separate aetiology [8]–[10]. Crucially, we know little, except for very recent work, about the nature and reason for delays involved between presentation of danger signs and arrival at hospital and/or provision of antimalarial treatment to children in Sub-Saharan Africa - an important basis for reducing this interval and improving child survival [11]–[13].

As the rectal artemisinins are now recommended as emergency pre-referral treatment for children who cannot take drugs by mouth, in settings where parenteral treatment is not immediately accessible [14], it is important to understand how these recommendations can be applied and integrated with existing patterns of managing and treating severe malaria. A study on local understanding, perceptions, treatment practices for signs and symptoms of severe malaria and barriers to immediate treatment of these symptoms was carried out prior to, and in preparation for, an intervention that provided early treatment of *non per os* children with rectal artesunate as emergency treatment for children with such symptoms at the community level in rural Tanzania. The intervention is ongoing and its results will be presented separately. This paper reports, therefore, the findings of the formative study.

## Materials and Methods

### Study area and population

The study was conducted in Mtwara Rural district in Mtwara region south of Tanzania, which is mainly inhabited by the Makonde ethnic group. The district has a total population of 212,019 and approximately half of the population (96,593) does not have access to health facilities in close proximity. Malaria transmission in the district is perennial and falciparum malaria is the leading cause of outpatient attendances (53%) as well as in-patient (39%) attendances, mostly under-five children. Health care services include a regional hospital, 4 health centres and 30 dispensaries. Dispensaries treat uncomplicated malaria among other illnesses and refer severe cases to health centre or hospital. Trained village health workers (VHW) and Traditional Birth Attendances (TBA) deliver community based health services.

A census of 67 out of 112 villages that do not have access to a dispensary or health facility in close proximity in Mtwara Rural district was conducted in 2004. Four rural communities/villages were purposively selected taking into consideration the geographic diversity of the district and accessibility to a health facility. Administratively, each village is divided in to 3–6 hamlets depending on the size of the village. The total population of the selected communities was 6915 of which 15.9% (n = 1099) were children under the age of 5 years (data from census).

### Data collection

Qualitative approaches consisting of key informant interviews (KII), focus group discussions (FGD) and in-dept interviews (IDI) were used. Interviews and discussions were held by Kiswahili speaking research assistants and a social scientist.

Using semi-structured and open-ended individual interviews, fifteen KII were involved in the study. The targeted respondents included health care providers (traditional birth attendants, village health workers, health workers and drug vendors), elders, opinion leaders/community leaders and parents of under-five children. The question guides for the KII directed them to record local diseases that could lead to death especially in children, local understanding of the signs and symptoms of malaria with special emphasis on severe illness suggestive of severe malaria, the underlying symptoms or illness-specific causes, patterns of symptom-specific actions and treatment seeking pathways by parent/guardians, factors causing delay in seeking care from health facilities and the decision making hierarchy at household level for seeking care outside the home. Local experience with use of rectal medicines was also investigated.

Through KII and participants of FGD, traditional healers who attend severely ill children in their communities were identified. In each of the four study villages one traditional healer was interviewed as the local specialist in treating children with symptoms associated (in the biomedical construct) with severe malaria. Traditional healer interviews were semi-structured and focused on exploring healers' descriptions of local illness and healing concepts, especially the associated Kimakonde terminologies, and treatment practices mentioned by community key informants and focus group participants

Using guided themes, 14 FGD of 6–8 participants per group were conducted with homogenous groups deliberately chosen to represent those most directly involved in providing care and making treatment decisions for severely ill young children. Mothers, who currently had a child under the age of five years at the time of the study (young mothers' group), women over 45 years, who, in their roles as mothers, grandmothers, mothers-in-law, aunties, etc, exerted influence over mothers of under-fives (old mothers' group), fathers who had a child under the age of 5 years at the time of the survey and (fathers' group) and health workers of Kitere and Mahuranga Health Centres (health workers' group) serving the study communities were targeted. The content of FGD guide was similar to that provided to the key informants. Each FGD session took an average of 45 minutes.

For the in-depth interviews (IDI), 12 mothers of under-five children whose children were admitted to the Regional Hospital with a diagnosis of severe malaria (altered consciousness, coma, convulsions, hypoglycaemia, difficulty in breathing, severe anaemia, prostration) were interviewed to obtain clinical history and treatment-seeking behavior for the illness. The interviews focused on (i) mother's recognition of signs and symptoms with which the child presented during the actual illness, (ii) mother's description of sequences of symptom presentations and of actions taken in seeking care during the child's present episode. Each IDI lasted on average 40 minutes. This group of mothers is referred to as “hospital mothers” in the paper.

In addition to the qualitative survey, monthly surveillance of febrile episodes with or without danger signs or symptoms/signs suggestive of severe malaria (failure to feed, vomiting, extreme weakness, convulsions, coma) among under-five children in the study communities were recorded for 4 months using quantitative methods. All households with children under the age of five in each community were mapped during the census and a surveyor was recruited for each hamlet and trained to conduct a monthly fever and danger-sign survey using a structured questionnaire.

The interviews were held in Kswahili, tape recorded and transcribed. The unit of analysis for the KII and IDI was the individual and it was the whole group for the FGD. The data were analyzed manually by applying codes within the structure of the thematic guide. The data were then structured into categories and put into a grid before identifying patterns, consensus, variation, contradiction and associations between variables [15]. The community surveillance data were entered using Epidata software and analyzed using STATA (version 9.2) programme. The findings generated through the different methods were integrated and triangulated on presentation. In addition, findings from the non health worker KI was triangulated with health care providers

### Ethical approval

The study was approved by the Medical Research Co-coordinating Committee of the National Institute for Medical Research (NIMR), Tanzania. Permission was obtained from the regional, district and village authorities. Verbal consent was obtained from the participants.

### Data analysis

The structure of study findings first considers symptom recognition, disease concepts and aetiology of two main perceived leading causes of death in children, malaria and convulsions. The second section discusses patterns of treatment for severely ill children with an emphasis on symptoms associated with malaria, convulsions and other bio-medically recognized symptoms of severe malaria. In the first section, information from the focus group discussions and from community key informant interviews forms the bulk of the data considered supplemented by and triangulated with data from traditional healer interviews, health care provider FGDs) and the quantitative survey data. In the section that follows, data from the interviews with hospital mothers are introduced and considered in conjunction with and in relation to data collected from all other study sources.

## Results

The characteristics of the participants in the KII, FGD groups and IDIs are presented in Table 1. The most common health problems affecting children were malaria, diarrhoeal disease, respiratory problems and convulsions (Table 2) while malaria and convulsions were the first and second leading causes of death among children. Only one of four traditional healers interviewed (TH from Msakala) listed malaria among the diseases leading to death of children.

**Table 1:**
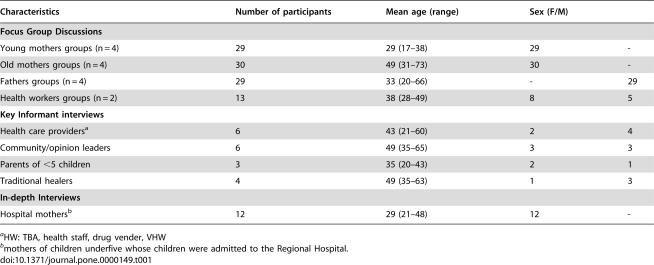
Sociodemographic profile of the study participants.

Characteristics	Number of participants	Mean age (range)	Sex (F/M)	
**Focus Group Discussions**				
Young mothers groups (n = 4)	29	29 (17–38)	29	-
Old mothers groups (n = 4)	30	49 (31–73)	30	-
Fathers groups (n = 4)	29	33 (20–66)	-	29
Health workers groups (n = 2)	13	38 (28–49)	8	5
**Key Informant interviews**				
Health care providers^a^	6	43 (21–60)	2	4
Community/opinion leaders	6	49 (35–65)	3	3
Parents of <5 children	3	35 (20–43)	2	1
Traditional healers	4	49 (35–63)	1	3
**In-depth Interviews**				
Hospital mothers^b^	12	29 (21–48)	12	-

a HW: TBA, health staff, drug vender, VHW

b mothers of children underfive whose children were admitted to the Regional Hospital.

**Table 2:**
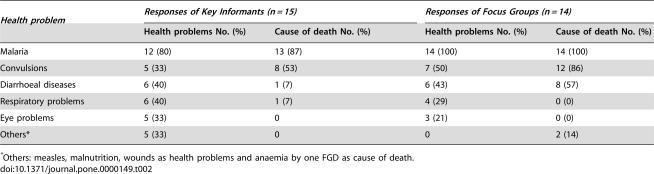
Key Informants' and Focus Groups' report on main health problems and leading cause of death in children in the study communities.

*Health problem*	*Responses of Key Informants (n = 15)*		*Responses of Focus Groups (n = 14)*	
	Health problems No. (%)	Cause of death No. (%)	Health problems No. (%)	Cause of death No. (%)
Malaria	12 (80)	13 (87)	14 (100)	14 (100)
Convulsions	5 (33)	8 (53)	7 (50)	12 (86)
Diarrhoeal diseases	6 (40)	1 (7)	6 (43)	8 (57)
Respiratory problems	6 (40)	1 (7)	4 (29)	0 (0)
Eye problems	5 (33)	0	3 (21)	0 (0)
Others*	5 (33)	0	0	2 (14)

* Others: measles, malnutrition, wounds as health problems and anaemia by one FGD as cause of death.

### “Malaria” terminology and disease concept

The local terminologies for common illnesses are summarized in Table 3. The study found that the term ‘malaria’ was widely used in Kiswahili discourse to describe fever illnesses. The Kiswahili term *homa*, commonly translated as ‘fever,’ and *homa kali* ‘fierce fever’ were terms used interchangeably by the respondents with the word malaria. Although the term *homa* sometimes has other implications in the wider taxonomy of disease, *homa* and *homa kali* always carried a primary association with *mwili joto* or ‘hot body’ (also: *mwili kuchemka*, ‘boiling body’). Both terms, malaria and homa, were reported to be current and carry the same meaning in the vernacular Kimakonde as well as local Kiswahili discourses. Another term used to describe malaria was *homa ya maleria*.

**Table 3:**
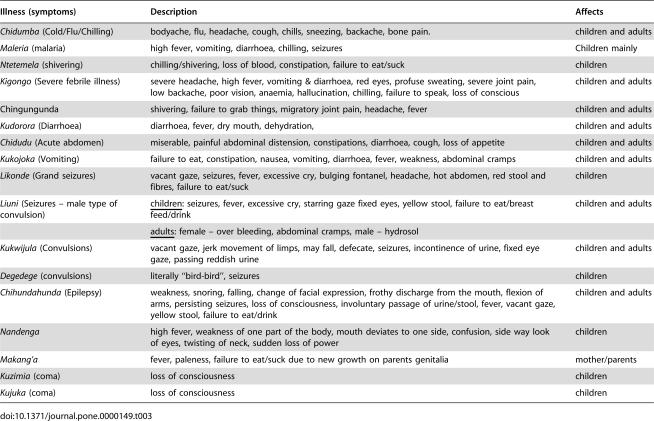
Local terminology of common illnesses in the study community and their descriptions

Illness (symptoms)	Description	Affects
*Chidumba* (Cold/Flu/Chilling)	bodyache, flu, headache, cough, chills, sneezing, backache, bone pain.	children and adults
*Maleria* (malaria)	high fever, vomiting, diarrhoea, chilling, seizures	Children mainly
*Ntetemela* (shivering)	chilling/shivering, loss of blood, constipation, failure to eat/suck	children
*Kigongo* (Severe febrile illness)	severe headache, high fever, vomiting & diarrhoea, red eyes, profuse sweating, severe joint pain, low backache, poor vision, anaemia, hallucination, chilling, failure to speak, loss of conscious	children and adults
Chingungunda	shivering, failure to grab things, migratory joint pain, headache, fever	children and adults
*Kudorora* (Diarrhoea)	diarrhoea, fever, dry mouth, dehydration,	children and adults
*Chidudu* (Acute abdomen)	miserable, painful abdominal distension, constipations, diarrhoea, cough, loss of appetite	children and adults
*Kukojoka* (Vomiting)	failure to eat, constipation, nausea, vomiting, diarrhoea, fever, weakness, abdominal cramps	children and adults
*Likonde* (Grand seizures)	vacant gaze, seizures, fever, excessive cry, bulging fontanel, headache, hot abdomen, red stool and fibres, failure to eat/suck	children
*Liuni* (Seizures – male type of convulsion)	children: seizures, fever, excessive cry, starring gaze fixed eyes, yellow stool, failure to eat/breast feed/drink	children and adults
	adults: female – over bleeding, abdominal cramps, male – hydrosol	
*Kukwijula* (Convulsions)	vacant gaze, jerk movement of limps, may fall, defecate, seizures, incontinence of urine, fixed eye gaze, passing reddish urine	children and adults
*Degedege* (convulsions)	literally “bird-bird”, seizures	children
*Chihundahunda* (Epilepsy)	weakness, snoring, falling, change of facial expression, frothy discharge from the mouth, flexion of arms, persisting seizures, loss of consciousness, involuntary passage of urine/stool, fever, vacant gaze, yellow stool, failure to eat/drink	children and adults
*Nandenga*	high fever, weakness of one part of the body, mouth deviates to one side, confusion, side way look of eyes, twisting of neck, sudden loss of power	children
*Makang'a*	fever, paleness, failure to eat/suck due to new growth on parents genitalia	mother/parents
*Kuzimia* (coma)	loss of consciousness	children
*Kujuka* (coma)	loss of consciousness	children

The terms *kigongo* and *chidumba* were mentioned as Kimakonde equivalents of malaria/*homa* in several FGD and by a small number KII, although both traditional healers and health centre staff who mentioned *kigongo* and *chidumba* categorized them as separate illnesses affecting both children and adults: *kigongo* severe febrile illness and *chidumba* as flu/cold.

*Likonde* (a Kimakonde term) was commonly used to describe convulsion illnesses affecting children although the respondents were also familiar with the term *degedege,* a Kiswahili term for convulsion illnesses in children. Other local terms for convulsion illnesses mentioned included *liuni* (male type of likonde of yellow stool and of greater severity) and *Kukwijula* (seizure illness related to likonde but also affects adults).

### Recognition of symptoms of malaria

There was substantial agreement between all categories of participants on the main symptoms associated with malaria/*homa* with fever, vomiting, shivering, confusion/delirium and headache being the most commonly mentioned symptoms (Table 4). High fever was defined as increased body heat, *mwili joto* or *mwili kuchemka*, sometimes as hot abdomen (*tumbo kuwaka joto kali*) or hot head (*kichwa joto*) with many informants also mentioning chills and/or shivering (*kutetemeka*). A few stated that relatively mild raised temperatures were normally interpreted as a sign of malaria, with very high fevers attributed to other illnesses, particularly to those associated with evil spirits or witchcraft. Through the monthly community surveys of underfive morbidity, a total of 1054 fever episodes were reported among the 1099 under-five children during the period of March–June 2005, the peak of malaria season, indicating that febrile illness in children under 5 years is very common in the area.

**Table 4:**
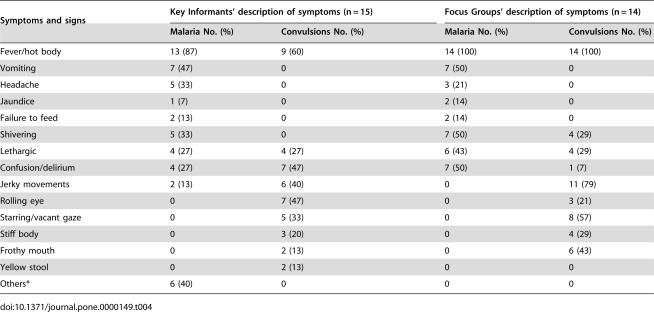
Key Informants' and Focus Groups' description on symptoms and signs of malaria and convulsions, leading cause of death in children in the study communities.

Symptoms and signs	Key Informants' description of symptoms (n = 15)		Focus Groups' description of symptoms (n = 14)	
	Malaria No. (%)	Convulsions No. (%)	Malaria No. (%)	Convulsions No. (%)
Fever/hot body	13 (87)	9 (60)	14 (100)	14 (100)
Vomiting	7 (47)	0	7 (50)	0
Headache	5 (33)	0	3 (21)	0
Jaundice	1 (7)	0	2 (14)	0
Failure to feed	2 (13)	0	2 (14)	0
Shivering	5 (33)	0	7 (50)	4 (29)
Lethargic	4 (27)	4 (27)	6 (43)	4 (29)
Confusion/delirium	4 (27)	7 (47)	7 (50)	1 (7)
Jerky movements	2 (13)	6 (40)	0	11 (79)
Rolling eye	0	7 (47)	0	3 (21)
Starring/vacant gaze	0	5 (33)	0	8 (57)
Stiff body	0	3 (20)	0	4 (29)
Frothy mouth	0	2 (13)	0	6 (43)
Yellow stool	0	2 (13)	0	0
Others*	6 (40)	0	0	0

The quantitative survey revealed that 27% (n = 281) and 22% (n = 232) of the reported fever episodes among under-fives in the study communities were accompanied by vomiting and failure to drink/suck/eat, respectively. Vomiting (*kutapika*) especially when frequent was listed as a symptom of malaria/*homa*, but consistently not of *degedege/likonde*:

*“(a) child is not supposed to vomit frequently, it can be either two or three times, but (if it is) more than that the parent realizes that the child has been attacked (by malaria sickness)” (KII father, Kihamba)*

*“(b) for the symptoms of malaria, I have to start by looking at what happened to my child. It started with crying, later on vomiting, headache, then colicky abdomen. I rushed to the hospital, the doctor said that the child had malaria and that due to vomiting intravenous fluids were needed, but even before one unit was started — (the child died)” (KII, Religious Leader, Kihamba)*

Lethargy (*Kunyong'onyeka/kulegea*) was mentioned by both categories of respondents as a symptom associated with malaria/*homa* and/or *degedege/likonde* (Table 4) and was found to be the leading symptom (28%, 291/1054) accompanying the fever episodes reported among under-fives in the study communities. It is seen as indicator of severe illness:

*“The difference between mild and severe malaria: severe malaria goes to the head, develops into high body temperature which spreads to the whole body. Then the child becomes lethargic, too weak to stand up and fails to make commands/requests. Then fails to eat and suck. Even fails to hold the breast” (KII shopowner, Kawawa)*

A child suffering from loss of appetite, paleness, inability to eat/drink/suck coupled with fever usually raises suspicion of *makang'a*, a condition suffered by adults. Most respondents described *makang'a* as growths on the genitalia (usual biomedical interpretation: warts) although traditional healers mentioned that such growths might also be detected inside the nose or ear. Whether it was their location or seriousness was not probed.

*Degedege/likonde* equivalent to convulsions was reported to be the second most frequent illness leading to death in the study communities (Table 2). The community surveys showed a prevalence of 10% (103/1054) of *degedege/likonde* among the reported under-fives' fever episodes. All categories of respondents reported that this condition affects young children between the age of weaning or crawling and five to six years of age. Care-givers gave a detailed description of *degedege/likonde* episode:

*(a) “the child has jerky movements, then (…) falls down, or if s/he doesn't fall down s/he can look with a staring gaze or looks as if s/he has been attacked by an evil spirit and thereafter the eyes roll side to side while the black spot in the centre looks pale” (KII young father, Chemchem Village).*

*(b) “the child starts with high fever (mwili joto), seizure (kukwijula), followed by fixed gaze” (FGD Young mother, Kawawa Village)*

Two types of convulsions with fever were locally recognized, male (*likonde dume*) and female (*likonde jike*). The male type, named by many as *liuni*, was described as more severe and likely to result in serious sequelae such as mental retardation or death. The passage of yellow stools was frequently mentioned as a symptom of convulsions, most often (but not exclusively) associated with the more severe ‘male’ convulsions.

Some informants consistently differentiated *degedege/likonde* from a chronic seizure disorder named as *chihundahunda/kifafa* (epilepsy), which was understood to affect adults as well as older children; however, the distinction between the two presentations was not always made; a number of informants stated that improperly treated *degedege/likonde* could progress to *chihundahunda/kifafa* and traditional healers implicated the same evil spirits as responsible for *degedege/likonde* in the later development of *chihundahunda/kifafa*.

Changes in mood or mental status were widely reported among the symptoms of malaria/homa, including mental confusion (*kuchanganyikiwa*), irritability and delirium (*kuweweseka*). *Kushtuka* (jumpiness, startling, sudden jerky movements) was a frequently recognized symptom in convulsing children although sometimes mentioned in connection with malaria, as observed by an older mother:

*“(with malaria) the child starts to rise in body temperature, then she may babble deliriously (talk irrelevancies) and startle easily, have jerky movements (kushtuka)” (KII, grandmother, Msakala).*

One traditional healer (Msakala village), who mentioned loss of consciousness, listed it among the symptoms of *Kigongo* (severe febrile illness). The presentation of a child in coma was reported as an infrequent condition:

*“That problem (loss of consciousness) does not (usually) happen (in this village), but if it did happen we would pour water over the patient then send (him/her) to the traditional healer” (KII Village Executive Officer, Msakal)*

This is supported by the quantitative survey which revealed that only 1% (14/1054) of the reported fever episodes was reported to have been accompanied by altered consciousness including coma. Respondents sometimes mentioned links between loss of consciousness and convulsions:

*“Coma (kuzimia) - occurs in children with convulsions and locally is known as kujuka. It is like dying but the one who is dead never comes up again but those in coma regain consciousness later on” (KII TBA, Kawawa ).*

### Perceived severity, cause and progression of disease

Although malaria/*homa* was the most frequently mentioned illness that could lead to death in children, symptoms suggestive of severe malaria were generally not categorized as malaria by study respondents. Coma (*kuzimia/kujuka*) was mentioned by only one respondent as a possible symptom of severe malaria; when other respondents were prompted to discuss coma, they asserted that coma was not associated with malaria. Moreover, the onset and progression of malaria/*homa* was generally seen as gradual. Any rapid death in the communities was viewed with suspicion, and not necessarily associated with malaria:

*“When the child is taken by god after (only) two or three days of illness, they (family members) say ‘no, this can't be malaria, probably it is sorcery, it couldn't be malaria affecting (the child) in such a (short) period of illness’ (KII teacher II, Msakala).*

The onset of degedege/likonde was variously described as gradual, with initial signs including high fever and excessive crying, or as very rapid, with the first symptom being a seizure:

*“A child will be playing, eating as usual, and all of a sudden develops jerky movement (kushtuka), the whites of the eyes will then protrude. In hospital this condition is called degedege” (KII, TBA, Kawawa).*

Thirty-six percent of the FGDs and 47% of the Key Informants reported that *degedege/likonde* was caused - or believed to be caused - by supernatural agencies; often evil spirits (*subiani or chipatupatu*). A traditional healer stated:

*“convulsions are caused by a demon known as ‘kufali’ ” (KII traditional healer, Kawawa Village)*

This condition was also associated with sorcery/witchcraft or god's curse,’ or ‘god's plan’ as noted by a village health worker:

*“Degedege itself – the experts (traditional healers) say it is caused by evil spirit “shetani”. Sometimes they tell you the child is bewitched” (FGD young mothers, Kawawa)*

While supernatural causation was prominent in the discourse about *degedege/likonde*, 57% of the FGDs (especially the women) and 40% of the key informants related it to malaria. A few respondents were specific in describing *degedege/likonde* as advanced or late-stage malaria, as in the following statement made by a key informant in Msakala:

*“Convulsion (degedege) is caused by mosquitoes; in other words, we can say it is matured (form of) malaria (malaria iliyokomaa)” (KII Teacher 1, Msakala).*

‘Malaria’ and illnesses caused by ‘evil spirits’ were often explicitly described as mutually exclusive, though it was clear that differentiating between the symptoms of the two could be difficult and might require specialist knowledge:

*“There are some people (diviners/traditional healers) who can tell you whether the disease is malaria or it is (caused by) an evil spirit” (KII, Grandmother, Kihamba Village).*

There were no health facilities, private dispensaries, drug shops or pharmacies situated within any of the study villages, although distance to the nearest Primary Health Care facility varies from 3–10 km. Small general goods shops (*duka*) are abundant in the villages and many of them stock and sell antipyretics/analgesics. No evidence of retail outlets selling antimalarials or other medicines was available in any of the study villages. There were traditional healers with different specialties: (i) healers ‘of books’ are scriptural healers whose healing practices centre around the use of books and writing and are main providers of charms to prevent degedege/likonde; (ii) healers ‘of evil spirits’ are mediums and diviners who contact and exorcise or appease the malevolent spirits responsible for causing a patient's ill health; (iii) herbalists use herbal remedies and are the main traditional providers of treatment for convulsing children in the study villages; (iv) witchcraft: secretive agents of intentional harm, is greatly feared.

All the FGD participants and 93% of the Key Informants mentioned the health facility as the resort of treatment for malaria/*homa*. The initial management of fever is mainly through the administration of drugs purchased from the shops or through sponging. Patients whose symptoms deteriorate are taken to a health facility if it is normal malaria. However, those with “*homa kali* – fierce fever” would be taken to the traditional healer:

*“At the traditional healer, they write using special ink, bathing the patient so as to remove evil spirits, they also smear medicine and tie charms (hirazi) to the patient” (KII, VHW, Msakala)*

Lethargy was said to increase the care-giver's level of concern and might therefore contribute to a decision to seek care outside the home; however, respondents did not appear to associate this symptom more with one type of treatment system than another.

Along with high fever, several respondents spoke of vomiting (*kutapika*) and/or failure to eat/drink/suck as symptoms of malaria/*homa* that did not necessitate outside treatment on their first occurrence but which raised the level of care-giver's concern; however, little information was conveyed about how “vomiting” or “failure to eat/suck” were managed. A few respondents mentioned trying to tempt a sick child to eat by offering different types of food, or forcing liquids on the patient, but in general vomiting or failure to eat/suck seemed to be often ‘bundled’ with other likely more alarming symptoms, than as events of particular importance in themselves.

Seven of the 12 mothers with currently admitted children mentioned vomiting on the first day of their child's current illness (Table 5) and six of them had claimed that they had administered medicine by mouth on that same day (3 antipyretics only and 3 with antimalarials dispensed by clinic). The timing of vomiting in relation to administration of oral medication was not specifically probed. However, one mother reported that her child had vomited the tablets.

**Table 5:**
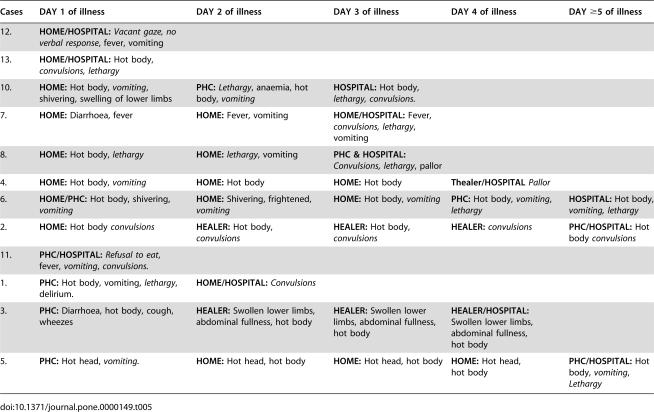
Care-seeking patterns for children (n = 12) with severe malaria admitted at Mtwara Regional hospital.

Cases	DAY 1 of illness	DAY 2 of illness	DAY 3 of illness	DAY 4 of illness	DAY ≥5 of illness
12.	**HOME/HOSPITAL:** *Vacant gaze, no verbal response*, fever, vomiting				
13.	**HOME/HOSPITAL:** Hot body, *convulsions, lethargy*				
10.	**HOME:** Hot body, *vomiting*, shivering, swelling of lower limbs	**PHC:** *Lethargy*, anaemia, hot body, *vomiting*	**HOSPITAL:** Hot body, *lethargy, convulsions.*		
7.	**HOME:** Diarrhoea, fever	**HOME:** Fever, vomiting	**HOME/HOSPITAL:** Fever, *convulsions, lethargy*, vomiting		
8.	**HOME:** Hot body, *lethargy*	**HOME:** *lethargy*, vomiting	**PHC & HOSPITAL:** *Convulsions, lethargy*, pallor		
4.	**HOME:** Hot body, *vomiting*	**HOME:** Hot body	**HOME:** Hot body	**Thealer/HOSPITAL** *Pallor*	
6.	**HOME/PHC:** Hot body, shivering, *vomiting*	**HOME:** Shivering, frightened, *vomiting*	**HOME:** Hot body, *vomiting*	**PHC:** Hot body, *vomiting*, *lethargy*	**HOSPITAL:** Hot body, *vomiting,* *lethargy*
2.	**HOME:** Hot body *convulsions*	**HEALER:** Hot body, *convulsions*	**HEALER:** Hot body, *convulsions*	**HEALER:** *convulsions*	**PHC/HOSPITAL:** Hot body *convulsions*
11.	**PHC/HOSPITAL:** *Refusal to eat*, fever, *vomiting*, *convulsions.*				
1.	**PHC:** Hot body, vomiting, *lethargy*, delirium.	**HOME/HOSPITAL:** *Convulsions*			
3.	**PHC:** Diarrhoea, hot body, cough, wheezes	**HEALER:** Swollen lower limbs, abdominal fullness, hot body	**HEALER:** Swollen lower limbs, abdominal fullness, hot body	**HEALER/HOSPITAL:** Swollen lower limbs, abdominal fullness, hot body	
5.	**PHC:** Hot head, *vomiting.*	**HOME:** Hot head, hot body	**HOME:** Hot head, hot body	**HOME:** Hot head, hot body	**PHC/HOSPITAL:** Hot body, *vomiting*, *Lethargy*

Treatment of makang'a (abnormal tissue growths mainly on the genitalia) often led to consultation with traditional healers to examine and treat them. Treatment of the child was affected by excising the growths found on the parent. A traditional healer stated that without such treatment the entire family was vulnerable to the curse and the child was likely to die. One of the hospital mothers (N^o^ 3) mentioned makang'a as the reason she had sought treatment from a traditional healer before taking her child to the regional hospital. It was the child's paternal grandparents who had raised the possibility of makang'a and on day two of the illness they decided that the mother should be examined and treated by the traditional healer. The child's failure to improve following this treatment was the reason given for taking the child to the regional hospital two days later.

A few respondents mentioned measures taken in the home for coma, where care-givers might attempt to rouse the patient from coma before taking him/her to the traditional healer. This usually involved dousing the patient with water, as in the following typical response:

*“… if it did happen we would pour water over the patient then send (him/her) to the traditional healer” (KII Village Executive Officer, Msakala).*

A traditional healer (Msakala village) who mentioned loss of consciousness as a symptom of *Kigongo* acknowledged using brewed herbal tea as well as spraying a herbal preparation over the patient's nose. None of the mothers of children currently admitted to hospital listed coma/loss of consciousness among the events occurring at any point in their children's illness.

For *degedege/likonde*, participants in all FGDs and 47% of the Key Informants pointed to the traditional healer as the first resort for convulsing children. Participants in the health care providers' focus groups confirmed the practice but dissociated themselves from it. As one health worker quoted:

*“With degedege many rush to traditional healer. They don't believe that it can be treated at hospital” (FGD Mahuruga Health Centre)*

Forty-percent of the Key Informants and 42% of the focus groups (young and old mothers) mentioned a number of home treatments derived from traditional healing practices, which is initiated at home on an emergency basis when there was a significant barrier (e.g. night time). Smoking the convulsing child using elephant dung was a commonly mentioned practice:

*“You get elephant dung, you heat it in a small piece of pot and the smoke goes through to the child, and, god willing, the seizures can be alleviated” (KII auntie, Msakala)*

The presenting symptoms and corresponding treatment pathways for 12 children with severe malaria admitted at Mtwara hospital are summarized in Table 5. Three of the twelve hospital mothers acknowledged having “smoked” the children at home as the first or second treatment attempted. Several respondents, including traditional healers, also mentioned mixing the elephant dung with marijuana/bhangi before burning. Other home used traditional remedies included (i) smearing, bathing or fanning the convulsing child with local herbs, use of substance with offensive odor such as garlic, plant root (*nkuhu*). Dousing the convulsing child with cold water, urinating on or immersing the child in the sea water were usually described as emergency measures rather than complete treatments. Only one of the twelve hospital mothers mentioned dousing or immersing her convulsing child in water.

Most respondents identified first resort to the traditional healer as the prevailing norm for treatment of convulsing (/*degedege/likonde*) children even for those who associated convulsions with malaria:

*“Convulsions are caused by mosquitoes and in other words we can say it is a matured malaria (malaria iliyokomaa)…..in children convulsions are mostly treated by traditional healer” (KII teacher I, Chemchem)*

Rituals involving smoking, fanning, smearing, therapeutic marks on skin or skin incisions, and preparation of herbal potions were all associated with traditional healers' treatment of convulsing children. Key informants and FGD participants provided detailed descriptions of such treatment practices, as captured in the quotes below:

*“(The healer) takes a nyungo mat made like a big bowl and uses it as a fan, laying down the child and fanning him/her on the head until the body cools down, then (s/he) takes certain leaves, crushes them and uses them to bathe the child, then leaves the child to sleep” (FGD young mother, Kawawa).*

*“For the child who eats, the medicine is mixed with porridge; for the child who is unable to eat they do therapeutic marks on the skin (chale) or smear the child with medicines on the mother's breast” (VHW, Msakala)*

Half of the FGDs and 20% Key Informants positioned the traditional healer as a first-stage treatment for *degedege/likonde*, an act followed by visits to clinics or hospitals once the child was ‘over the crisis’:

*“if (the child) gets better you take her/him to the hospital” (FGD Old mothers, Kawawa)*

Of the 12 children admitted at hospital, 58% were reported to have had convulsions. Although many mothers had used traditional medicine either as home remedies (n = 5) or at traditional healers (n = 1), they also used biomedical treatment, hence the eventual resort to hospital, and admission of the child. The amount of time spent at the traditional healers was decided by the healer and was not seen as ‘negotiable’ by the care-givers and could vary from few hours to days:

*“They don't stay (at the traditional healer's place) for a long time. It may be for one hour or more and then they recover. And our traditional healer here in this place treats every type of disease” KII (Grandmother, Kihamba).*

*“At the traditional healer (you stay) until he declares that he has failed to treat. There is therefore no specific amount of time” (KII VHW, Msakala)*

Three of the twelve hospital mothers, those who had taken more than three days to seek care since the onset of symptoms, reported consultation with traditional healers with only one for convulsions. Several respondents described from personal experience or stated as a general truth that traditional healers retained patients in their care for lengthy periods even when treatment appeared to be failing. A teacher in one of the communities noted that

*“There is no traditional healer who can permit his/her patient to seek alternative treatment even if s/he finds the condition of the patient is not improving” (Teacher, Chemchem)*

A father remarked that:

*“(…) after one week the (child's) condition was not good so we asked permission to leave but the old lady (traditional healer) refused (…) eventually we had to give an amount of money which she required (equal to her fee had they continued for the full course of treatment) and then we left” (FGD Fathers, Msakala)*

In contrast to malaria/*homa*, for which no preventive measures were described, numerous informants mentioned methods of protecting children from *degedege/likonde* before, during or after an episode of the condition:

*“When the child is born there are some protective measures which should be done. Local herbs are applied to pierced skin on the sides of the child and this will protect the child against likonde. If the parents did not perform this, the child will be affected by likonde” (father, Kihamba Village)*

*“When the child falls sick s/he is given a protective charm to wear and grows with it until it falls off” (Traditional Healer, 35 years, Kihamba Village)*

Participants in five FGDs from 3 communities believed that injections, given to a convulsing child, could be fatal. An older mother noted that

*“If you inject a convulsing child you increase the poisoning of that child. Convulsion has poison and injection too, so you will be poisoning the child suddenly, you will kill him/her. "( FGD Old Mothers, Kihamba)*

Despite beliefs about the dangers of injection use in convulsing child, some care-givers sought care from health facilities, sometimes directly contravening the advice of elders:

*“Let me talk. My child was suffering from convulsions and as usual my mother came. She told me that if I took the child to hospital I would come back without the child, but I told her that what she was talking about was out-of-date. And I took my child (to hospital) and on the second day he was all right” (FGD fathers, Kihamba).*

The three mothers who went to the regional hospital on the first day of their child's illness (Table 3) reported convulsions as the symptom that had triggered their decision to seek immediate care at the hospital, which implies that despite what appears to be prevalent beliefs regarding the correct treatment, households are tending to take decisions that they consider critical to their children's survival.

### Decision-makers

The majority of the key informants (73%) and participants of the FGDs (86%)stated that decisions to seek care for severely ill children were made by fathers. If the father of a child was not around, the mother or grandparent made the decision. Mothers and fathers both reported seeking advice and hands-on assistance from family elders and neighbours, especially when symptoms developed at night. The in-depth interviews with the hospital mothers confirm this pattern of decision-making: 5 cases by fathers, 2 cases by mothers, 2 cases by both parents and the remaining 3 by relatives. The decision making process was not further probed to establish whether the pattern seen for hospital admissions and described in interviews was due to financial control, distance or the severity of the illness.

### Barriers to seeking biomedical treatment

Spatial and temporal considerations dominated discussions on factors inhibiting access to formal health facilities. Transport from the village to the nearest health facility was largely by bicycle, and many respondents complained of inadequate access:

*“Today my child can become ill, I'll go to my neighbor to ask for the bicycle and he will tell me that his bicycle is out of order [not working]. I'll proceed to another one and the answer will be the same, and that is when you decide to go on foot” (KII Religious Leader, Kihamba, distance to nearest facility 5 km)*

In the rainy season roads were reported to be difficult or impassable in Kawawa, Chemchem, and Kihamba:

*“…during rains, you can't ride a bicycle, the road becomes muddy and walking takes a long time. So during rains, transport is really a problem (Teacher I, Chemchem, distance to nearest facility 8 km).*

Perceived severity was sometimes a key indicator for resort to the nearby traditional healer while less urgent cases (no clear immediate danger) taken to the health facility.

*“People go to the local traditional healer when the patient is seriously ill because the hospital is at a distance. He might want to take the patient to the dispensary but it is very difficult (because of distance), so (he feels) it is better to rush the patient to the nearby traditional healer who treats even evil spirits — he might be of help” ( KII shop owner, Chemchem).*

Uncertainty about the likely course of illness was also cited as a factor that could cause delay in seeking treatment. Thus respondents often spoke of the decision to seek care for ‘common’ symptoms such as high fever in terms of a care-giver's personal ability to “tolerate” the child's illness during a watch-and-wait period:

*“Some believe(child may get better without specialist care) others not” (FGD young mothers, Kawawa)*

*“some can tolerate (waiting) while others can not” (KII Grandmother, Msakala)*

*“I can't wait while the child is sick — I can't! My child is sick and I want him to get medicines!” (KII VHW, Kihamba)*

Night was a significant barrier to move out of the home, with respondents mentioning fear of wild animals and sense of unspecified dangers “after sunset” as factors that would effectively prohibit most people from venturing out until morning. Neighbors were the stand-ins for medical providers during night time hours, being called on for advice, reassurance, and practical help during long night vigils with sick children. One of the 12 hospital mothers mentioned fear of moving in the night alone as the main reason she had not taken her convulsing child to the hospital straight away, instead she called in the neighbors who assisted with emergency ‘first aid’ by smoking elephant dung and immersing the child in a bucket of water.

Cost of care was frequently raised to explain delays in accessing both traditional and biomedical treatment. In the case of treatment at the traditional healer's place, cost could cause a double delay: first as a barrier to accessing care from the traditional healer as the parents have to secure money to pay the service and, if treatment was initiated before the family had managed to ‘find’ the money to pay for it, as a barrier to leaving the traditional healer's place in order to seek hospital care:

*“At the traditional healer, payment is needed and if there is nothing in the house the mother will delay while trying to raise some money. If they happen to go to the traditional healer (without money) and be accepted, two things may happen. Mother and child will remain at the traditional healer's place while father is looking for money, or mother will have to leave some of her clothes (as surety) and come back to collect them when she gets some money” (KII Teacher I, Chemchem)*

Out of the 12 hospital mothers, 33% reported “lack of money” as the main reason for their delay in getting the child to hospital. Although consultations and treatment were free for under-fives as part of government policy, money was needed for transport, and also sometimes more directly for the treatment itself. Several FGD and key informant respondents reported unofficial costs associated with health facilities, often arising from a poor supply situation that resulted in patients having to purchase the needed medicines or equipment from private suppliers:

*“You can reach there (dispensary) and find that one syringe costs five hundred shillings (0.4 US$), and if you don't have that money it means you can't get treatment” (KII Grandmother, Kihamba)*

Another potentially significant delay factor was seen in relation to the care-giver's lack of decision-making power or independent access to the required resources (mainly money), with many respondents mentioning the mother's need to wait for the father's permission before embarking on treatment:

*“(The) mother will not take a sick child to the hospital unless the father returns home. (…) Since the father may have many wives it is quite possible that the sick child is not from the household where the father slept that night. The child will not be taken to hospital until the father is consulted, “the child is sick, should we buy him an aspirin or take him to the hospital?” (KII VHW, Msakala).*

Two of the 12 hospital mothers stated the absence of the father to give permission or provide them with money was the main cause of delay in taking their sick children to hospital. It was also mentioned that conflicting treatment preferences between care-givers and decision-makers were a cause of delay: one hospital mother reported that although she had wanted to take her child to the hospital sooner, elderly members of the extended family had advised her to go to the traditional healer for *makang'a* treatment first.

Despite the many barriers and constraints people faced in seeking care from health facilities, 40% of the Key Informants and 36% of the FGDs stated that when a child was seriously ill such obstacles simply had to be overcome:

*“You can't stay idle waiting for rain to stop; you know how serious the child's fever is. (…) some have bicycles; others, like us, do not. Therefore it is possible that even ‘though it is midnight you carry the grandchild and off you go (on foot)” (Grandmother, Msakala)*

When respondents were asked if they had heard or had direct experience of rectal application of medicines, 86% of the focus groups and 67% of the Key Informants stated that they were familiar with some type of rectal medicine, either traditional or biomedical. These included rectal use of herbs for abdominal distention and *chidudu,* soap for constipation and diazepam for convulsions (at health facilities). All these respondents also thought that rectal medicine would be acceptable in their communities. The remaining respondents were either not familiar with the practice (4 Key Informants) or were not probed for response (2 FGD and 1 Key Informant).

## Discussion

This study provides new information on the pattern of danger signs in children in a hyperendemic area of malaria in Tanzania, and documents local perception and common treatment-seeking behavior related to each of these danger signs, or their combination. The communities' description of malaria/*homa* symptoms corresponded to the biomedical definition of uncomplicated malaria, and was considered treatable at health facilities. Although the description of *degedege/likonde* was consistent with the biomedical description of convulsions, a prominent feature of severe malaria, it was perceived as a separate disease associated with evil spirits and witchcraft, a perception noted in other parts of Tanzania [8], [10], [16] and elsewhere [9], [17]–[19]. Traditional healing - either at home or at a specialist traditional healer - was customary and the first option for the management of convulsions and altered consciousness, even if this was followed by hospital consultation. Such practice might cause delays to appropriate and effective treatment. The perception that an injection would be fatal for the convulsing children has been reported earlier [16] and might reinforce the use of traditional remedies. Coma (*kuzimia*) was rarely mentioned spontaneously and was referred to in the context of traditional healing. Lethargy was seen as a sign of severe illness and might provoke action outside the home. However vomiting and failure to eat/suck/drink raised concern but were not seen as indicators of severe illness; when combined with fever they were managed within the home. Vomiting was rarely mentioned in mother's accounts of symptoms; it was considered a mundane symptom, overshadowed by more disquieting symptoms that followed. The early occurrence of vomiting and failure to eat, while increasing anxiety, did so only in the presence of other more dominating symptoms and thus pointed away from the possible development of more serious complications associated with *degedege/likonde* instead of alerting care-givers to the increasing potential of severe disease.

Our findings reveal that the study communities were familiar with rectal application of medicine, mainly traditional medicine but also biomedical, an observation reported by Kaona and Tuba [11] from Zambia, arguing well for the potential acceptability of rectal artesunate.

Care-givers indicated that home treatments were often initiated as first aid before urgently seeking treatment from a traditional healer, or as ‘a holding measure’ undertaken if ‘expert treatment’ could not be accessed immediately (e.g. during the night). When the moment of crisis (the convulsion) was past and/or access barriers were removed, care-givers were seen as likely to seek follow-up care either at traditional healers or at a biomedical health facility or both. The fact that respondents who associated convulsions with malaria yet would seek care from traditional healers raises concern. Similar discrepancies between knowledge and treatment practice in convulsions have been reported from other parts of Tanzania [16].

The quantitative data in this study confirm findings elsewhere that febrile illness is common in children [20] but provide new data regarding the frequency of danger signs accompanying fever in the community - repeated convulsions, altered consciousness, repeated vomiting, lethargy, inability to eat/drink or suck. Each of these signs, separately or in combination, is part of the considerable overlap in clinical symptoms between malaria and pneumonia [21]–[23] that warrant urgent referral.

The WHO and UNICEF Integrated Management of Childhood Illness (IMCI) Guidelines developed in the mid-1990's to assist clinical discrimination between children needing urgent hospital management, and others needing symptomatic therapy recommend hospital referral for all children with danger signs including: convulsions, inability to drink, vomiting everything, unconscious or lethargy [24] as there is evidence at the facility level, that each hour of delay in identification and treatment of a child with elevated temperature and a danger sign is associated with increased risk of mortality [21], [25]. In a malaria endemic area, these danger signs would mark the evolution to severe disease, which is considered by most health authorities as a medical emergency requiring injectable treatment. The 2006 WHO Malaria Treatment Guidelines [14] have taken a step further by recommending the use of artemisinin-based suppositories for emergency treatment of patients who cannot take oral therapy by reason of their altered level of consciousness or protracted vomiting prior to their transfer to a health institution that can provide parenteral therapy.

While there is an extensive, coherent body of evidence collected for the purpose of developing, validating and refining IMCI facility guidelines [26], [27], [28] and renewed efforts quantifying the benefit of introducing facility based IMCI [29], [30], [31] there has been less focus on Household and Community-IMCI [32]. Thus the frequency, clinical presentation and mortality risks of different symptoms in the community constitute a neglected area of research despite an increasing awareness that appropriate and accessible care at the community level is crucial for achieving gains in child survival [33]. Consequently more research is required on interventions that could be introduced at the community level to improve community identification, treatment and referral of children with danger signs to improve child survival [34]. Finding the best way of delivering such interventions should also be a priority in malaria research [35].

The findings of the study have implications for the introduction of community-based interventions that improve management of severe disease, including the introduction of rectal artemisinins as pre-referral treatment at community level. Local communities' ability to recognize convulsions, altered consciousness and coma, their awareness about the risks of such conditions if not treated, and the fact that mothers are opting to go to a health facility even when children are convulsing might be facilitating factors for the introduction of artesunate suppositories. In addition, the practice of rectal administration of medicines by the study communities could be an added advantage. Biomedical options that are more convenient than traditional options for emergency care are easier to introduce- particularly where time, distance (dangers of movement at night), and money are involved. Emergency treatment with an artesunate-based suppository at the community level could enable the severely ill child to have treatment while the parents seek transport and funds for definitive care. However, poor discrimination of vomiting, lethargy and failure to feed as early signs of severe disease might call for health education with special emphasis on danger signs. Targeting such education to care-givers of young children, decision-makers especially men, advisors, formal and informal care providers might be a prerequisite for successful introduction of rectal artesunate as an emergency treatment. Traditional healers are part of the community and are easily accessible temporally and spatially and therefore might pose a major barrier to rapid use of the drug in a sick child and adhering to referral advice. Mechanisms involving traditional healers in delivering artemisinin-based suppositories, a medicine that holds great promise for Africa, should be explored.

### Methodological limitations

With the aim of facilitating the successful introduction of rectal artesunate through a well-grounded understanding of symptom recognition and treatment practices for “severe malaria,” the study collected qualitative data from several categories of people in different settings. The collection of data relied heavily on informants' reports of normative behavior in response to questions posed in a context far removed from the immediate stress and uncertainty of managing a child's actual illness which could lead to overstating perceived norms, underestimating, or downplaying the extent to which people normally deviate from them. There is a likelihood that the researchers themselves were perceived as representing the health service and had vested interests in the MoH/biomedical health system, which would tend to skew responses towards expression of greater acceptance of biomedical disease concepts and treatment practices.

Only “hospital mothers” whose children were out of immediate danger and pronounced as recovering - those for whom the move to hospital proved most rewarding – were interviewed. This might have contributed to a reporting bias with the likely tendency being to over-report recourse to biomedical therapies while under-reporting treatment delays or recourse to ‘traditional’ or other treatments. We also interviewed mothers who sought care at the highest level of the health system. This excludes those who did not make to the hospital for one reason or the other (a selection bias).

All interviews and focus group discussions were conducted in Kiswahili, the official language of Tanzania, while the vernacular language of the study area is Kimakonde. However, fluency in spoken Kiswahili is nearly universal among study participants who expressed themselves without apparent difficulty in all topic areas explored.
